# Genome-Wide Identification and Expression Analysis of NCED Gene Family in Pear and Its Response to Exogenous Gibberellin and Paclobutrazol

**DOI:** 10.3390/ijms24087566

**Published:** 2023-04-20

**Authors:** Jinming Liu, Xing Yuan, Shaowen Quan, Meng Zhang, Chao Kang, Caihua Guo, Zhongrong Zhang, Jianxin Niu

**Affiliations:** 1 Department of Horticulture, College of Agriculture, Shihezi University, Shihezi 832003, China; m15700983223@163.com (J.L.); 18899533862@163.com (X.Y.); qsw229@stu.shzu.edu.cn (S.Q.); 17590936852@163.com (M.Z.); kcshzu@163.com (C.K.); gch202205@163.com (C.G.); zhangzhongrong@stu.shzu.edu.cn (Z.Z.); 2 Xinjiang Production and Construction Corps Key Laboratory of Special Fruits and Vegetables Cultivation Physiology and Germplasm Resources Utilization, Shihezi 832003, China

**Keywords:** ‘Kuerle Xiangli’, genome-wide, *NCED* gene family, expression analysis

## Abstract

The 9-*cis*-epoxycarotenoid dioxygenase (*NCED*) is a key enzyme for the process of ABA synthesis that plays key roles in a variety of biological processes. In the current investigation, genome-wide identification and comprehensive analysis of the *NCED* gene family in ‘Kuerle Xiangli’ (*Pyrus sinkiangensis* Yu) were conducted using the pear genomic sequence. In total, nineteen members of *PbNCED* genes were identified from the whole genome of pear, which are not evenly distributed over the scaffolds, and most of which were focussed in the chloroplasts. Sequence analysis of promoters showed many *cis*-regulatory elements, which presumably responded to phytohormones such as abscisic acid, auxin, etc. Synteny block indicated that the *PbNCED* genes have experienced strong purifying selection. Multiple sequence alignment demonstrated that these members are highly similar and conserved. In addition, we found that *PbNCED* genes were differentially expressed in various tissues, and three *PbNCED* genes (*PbNCED1*, *PbNCED2*, and *PbNCED13*) were differentially expressed in response to exogenous Gibberellin (GA_3_) and Paclobutrazol (PP_333_). *PbNCED1* and *PbNCED13* positively promote ABA synthesis in sepals after GA_3_ and PP_333_ treatment, whereas *PbNCED2* positively regulated ABA synthesis in ovaries after GA_3_ treatment, and *PbNCED13* positively regulated ABA synthesis in the ovaries after PP_333_ treatment. This study was the first genome-wide report of the pear *NCED* gene family, which could improve our understanding of pear NCED proteins and provide a solid foundation for future cloning and functional analyses of this gene family. Meanwhile, our results also give a better understanding of the important genes and regulation pathways related to calyx abscission in ‘Kuerle Xiangli’.

## 1. Introduction

The plant hormone abscisic acid (ABA), considered as a universal stress hormone, plays a crucial role in the adaptation to biotic and abiotic stresses and in several physiological processes [1] such as seed development [2], root growth [3], plant responses to inhibition of water uptake, and drought stress [4,5]. The 9-*cis*-epoxycarotenoid dioxygenase (*NCED*) is a key enzyme in the ABA biosynthesis pathway [6]. *NCED* cleaves carotenoid C40 in plastids and produces ABA by catalysis of alcohol dehydrogenase and aldehyde oxidase [7].

The first *NCED* transcription factor was found in the maize *vp14* mutant [8]. Recently, several studies suggested that ABA results in decreasing the carotenoid content in juice sacs of citrus fruit by inducing its own biosynthesis, but the expression of *CitNCED2* and *CitNCED3* increases rapidly with ABA accumulation [9,10]. Similarly, the expression of *FaNCED1* plays a major role in ABA accumulation and the process of fruit ripening of strawberry [11,12]. Studies have shown that transcript levels of peach and grape NCED transcription factors regulate its fruit ripeness and senescence [13]. *OsNCED5* expression was found in all tissues and has similar functions to *OsNCED3* and *OsNCED4* in response to abiotic stress [14]. In *Arabidopsis* (*Arabidopsis thaliana*), overexpression of *AtNCED3* results in increased endogenous ABA accumulation and resistance to drought stress, as well as induced gene response of ABA [6]. Co-expression of *SgNCED1* and *ALO* genes increased tolerance to drought and chilling and induced expression of its related gene in the transgenic tobacco and stylo plants [15]. Real-time PCR analysis revealed *MpNCED2* were expressed continuously during the whole period of apple fruit development, while the expression of *MpNCED1* clearly declined to a steady low level in the mid–later period of fruit development [16]. Additionally, *NCED* is also an important candidate gene involved in the response to cold stress in panax ginseng (*P. ginseng*) and can act as an endogenous signaling molecule regulating cold stress and biosynthesis of *P. ginseng* [17].

The *NCED* gene family has been identified and studied in many plant species such as *Arabidopsis* [18], cotton [19], avocado [20], cowpea [21], kiwifruit [22], and grape [23]. Pears (*Pyrus* spp.) belong to the family Rosaceae, subfamily Maloideae, and genus Pyrus [24]. Pear is a major economic crop in the world [25]. However, the role of *NCED* genes has not been clearly identified in pear.

The pear genome was published in 2013, which provided an opportunity to reveal genome-wide *NCED* gene family [26]. In the present study, we used bioinformatics methods to identify *NCED* genes from the pear genome, and analyze the sequence features, evolutionary traits, tissue-specific expression levels, and expression patterns in response to exogenous Gibberellin (GA_3_) and Paclobutrazol (PP_333_). The results provide useful information for further functional investigations of the *NCED* gene family in pear.

## 2. Results

### 2.1. Identification and Analysis of PbNCED Genes

A total of 22 *PbNCEDs* were obtained by local BLASTP and Hidden Markov Model (HMM) analysis. After that, CDD and SMART were used to confirm the presence of the conserved RPE65 domain, which confirmed the existence of 19 putative *PbNCED* genes in the pear genome. Finally, the 19 *PbNCED* genes were named from *PbNCED1* to *PbNCED19* based on their scaffolds’ locations (Table S1).

The lengths of the *PbNCED* proteins ranged from 474 aa to 617 aa. The molecular weights of *PbNCED* proteins were between 53.92 kD and 69.13 kD. The predicted pI values of *PbNCED* proteins ranged from 5.61 to 7.64. All *PbNCED* proteins had typical secondary structures including Alpha helix, Beta turn, Extended strand, and Random coil. The *PbNCED* proteins contained the highest proportion of extended strands and the lowest proportion of β-folded structures except *PbNCED1* and *PbNCED2*. The result of subcellular localization showed that the *PbNCED* proteins were predicted to in the chloroplast, but a few *PbNCED* proteins possibly had a cytoplasmic, peroxisome, and mitochondria localization (Table S2).

### 2.2. Multiple Sequence Alignment and Phylogenetic Analysis of PbNCED Genes

**Figure 1:**
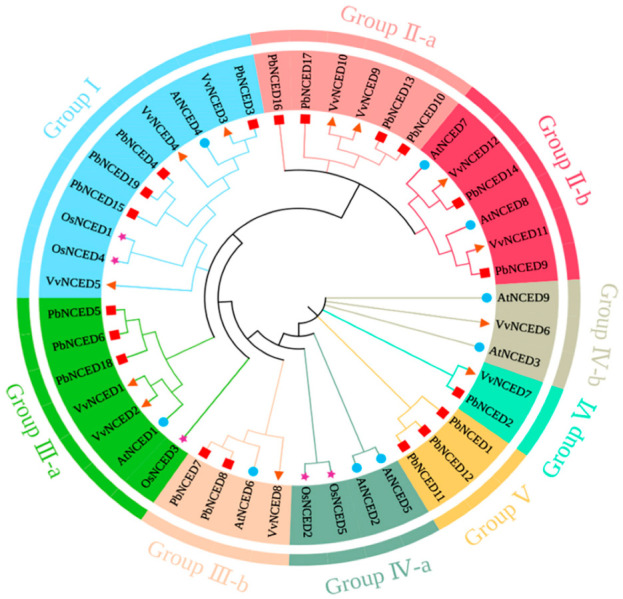
The phylogenetic relationship of the *NCED* gene family to *Arabidopsis*, rice, grape, and pear. Note: The blue solid dot represents *Arabidopsis* NCED protein, the purple solid pentagon represents rice NCED protein, the orange solid triangle represents grape NCED protein, and the red solid square represents pear NCED protein.

**Figure 2:**
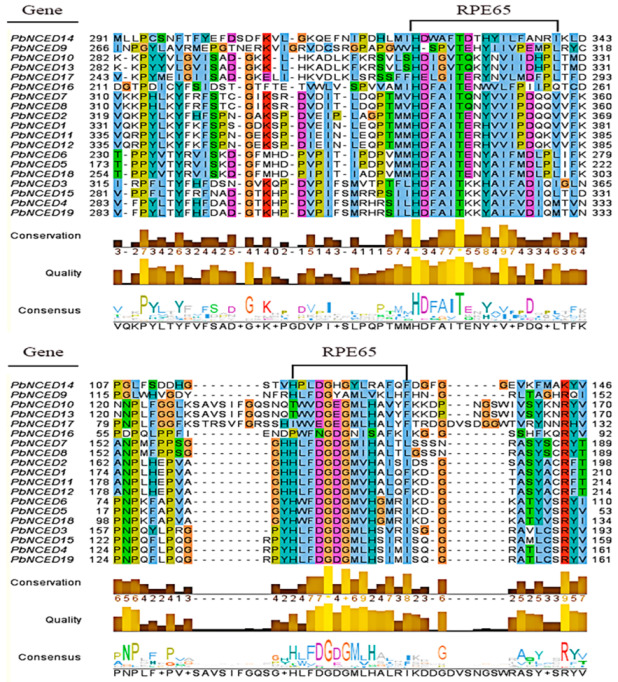
The RPE65 domain of *PbNCED* gene family protein.

To analyze the evolutionary relationships of *NCED* genes in pear, *Arabidopsis*, rice, and grape, a phylogenetic tree was constructed using MAGE-X software employing the neighbor-joining method (Figure 1). The phylogenetic tree showed that these *NCED* proteins could be classified into six groups, namely, Group Ⅰ, Group Ⅱ, Group Ⅲ, Group Ⅳ, Group Ⅴ, and Group Ⅵ, with well-supported bootstrap values. Additionally, Group Ⅱ, Group Ⅲ, and Group Ⅳ were further divided two subfamilies including Group Ⅱ-a, Group Ⅱ-b, Group Ⅲ-a, Group Ⅲ-b, Group Ⅳ-a, and Group Ⅳ-b, respectively. Nearly all groups included *PbNCEDs*, with the exception of GroupⅣ-a and GroupⅣ-b. The numbers of *PbNCEDs* in Group Ⅰ, Group Ⅱ-a, Group Ⅱ-b, Group Ⅲ-a, Group Ⅲ-b, Group Ⅴ, and Group Ⅵ were 4, 4, 2, 3, 2, 3, and 1, respectively. The *PbNCED* proteins had many conserved amino acids by multiple sequence alignment of *PbNCED* domains (Figure 2), especially histidine (H), glycine (G). and threonine (T). The results revealed that the sequences in the *PbNCED* domain were highly conserved.

### 2.3. Gene Structure and Motif Composition 

**Figure 3:**
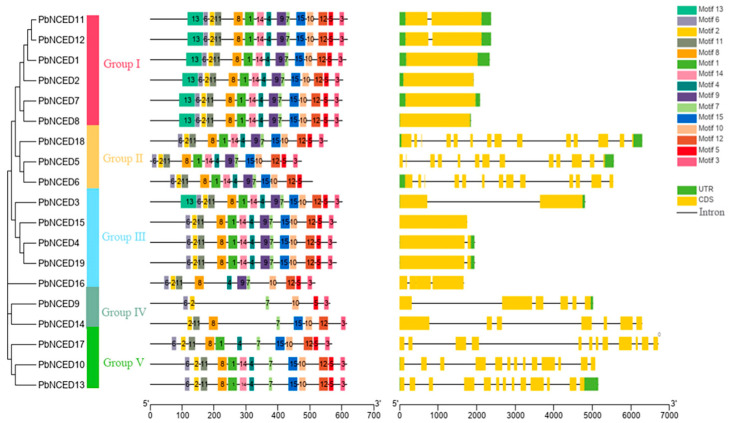
Phylogenetic relationship (**left**), conserved motif (**middle**), and gene structure (**right**) of *PbNCED* gene family protein.

To analyze the evolutionary relationships of *NCED* genes in pear, the phylogenetic tree was constructed using full length amino acid sequences. The 19 *PbNCEDs* were classified into five distinct subfamilies. The 15 conserved motifs were found among 19 *PbNCEDs* by MEME website, and the amino acid consensus sequence of each motif is listed in Supplementary Figure S1. The lengths of these conserved motifs ranged from 11 to 50 amino acids. Members of all *PbNCEDs* generally had more than seven motifs except for *PbNCED9*, which only contained six motifs. The similar motif arrangements among *PbNCED* proteins within subgroups indicated that the protein architecture is conserved within a specific subfamily. Further, in exploring the exon–intron structure characteristics of the *NCED* family in pear, we note that exons and introns existed in every *PbNCED* gene. The number of introns was the least in Group Ⅰ and Group Ⅲ, while Group Ⅱ, Group Ⅳ, and Group Ⅴ had a higher number of introns. These similar structural characteristics may be related to the functions of these genes in the pear genome (Figure 3).

### 2.4. Chromosomal Location, Gene Duplication and Synteny Analysis 

**Figure 4:**
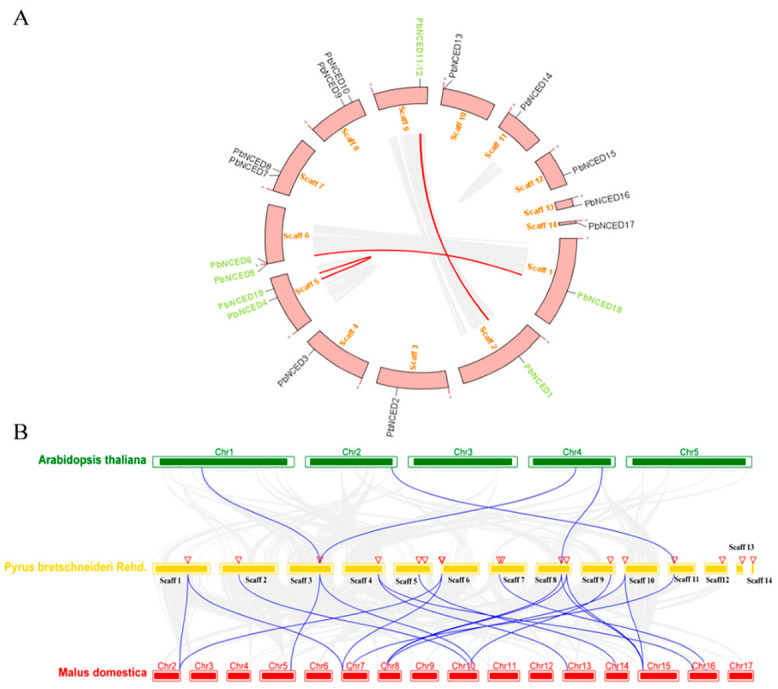
(**A**) Synteny analysis of *PbNCED* genes. Gray lines indicate all synteny blocks in the pear genome, whereas segmental duplicated homologous genes are indicated with red lines. (**B**) Synteny analysis of *NCED* genes between *Arabidopsis*, pear, and apple. The gray lines in the background indicate the collinear blocks with in pear and other plant genomes, while the blue lines highlight the syntenic *NCED* gene pairs. The chromosome number is shown at the top of every chromosome.

Physical locations of 19 *NCED* genes in pear were investigated by analysis of genomic distribution on scaffolds and were distributed on 14 pear scaffolds randomly (Figure 4A). In addition, scaffold5, scaffold6, scaffold7, and scaffold8 had two predicted *PbNCED* genes, but other scaffolds had only one. To better understand the evolutionary constraints acting on *PbNCED* gene family, the *Ks*, *Ka,* and *Ka/Ks* ratios of the *PbNCED* gene pairs were calculated to investigate the divergence time of the duplication blocks (Table S3). The five segmental duplication gene pairs were estimated to have occurred 31.40–84.984 million years ago. These duplicated genes were identified, and the Ka/Ks of all five pairs were <1, suggesting that the *PbNCED* gene family had experienced strong purifying selection and the slow evolution rate in pear. The CDS of *PbNCED4* and *PbNCED19* were the same so that cannot be used to calculate *Ka/Ks.*

To reveal the orthologous relationships of *NCED* genes on chromosomes between *Arabidopsis*, pear, and apple genomes, a comparative analysis was performed between *NCED* genes in *Arabidopsis*, pear, and apple by TBtools (Figure 4B). The four orthologous gene pairs were identified between *Arabidopsis* and pear. The 19 orthologous gene pairs were identified between pear and apple. The result indicated the close relationships between pear and apple, compared to *Arabidopsis* and pear. In addition, the three *NCED* genes (*PbNCED2*/*PbNCED9*/*PbNCED14*) were identified to have orthologous genes within the *Arabidopsis*, pear, and apple genomes simultaneously. These *NCED* genes might have evolved from the common ancestor in different plants.

### 2.5. Interaction Network and Cis-Regulatory Element Analysis

**Figure 5:**
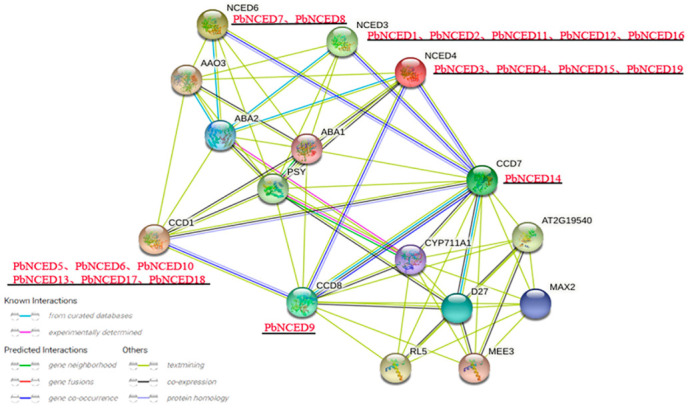
Protein interaction network of *PbNCEDs* constructed by referring to *AtNCEDs*. Note: The names of pear homologous proteins are highlighted in red.

The *PbNCED* proteins were constructed the protein–protein interaction network based on the interaction relationship of the homologous *NCED* proteins (*At*CCD1/*At*NCED3/*At*NCED4/*At*NCED6/*At*CCD7/*At*CCD8) in *Arabidopsis* (Figure 5). The *NCED* proteins played an important role in *Arabidopsis*. For example, *At*NCED3 played a major role in ABA synthesis under drought stress [6]; *At*NCED6 were involved in ABA synthesis during seed development [27]; and *At*CCD1, *At*CCD7, and *At*CCD8 were involved in carotenoid metabolic pathways [28]. The results illustrate that the *PbNCED* family proteins might have similar functions.

**Figure 6:**
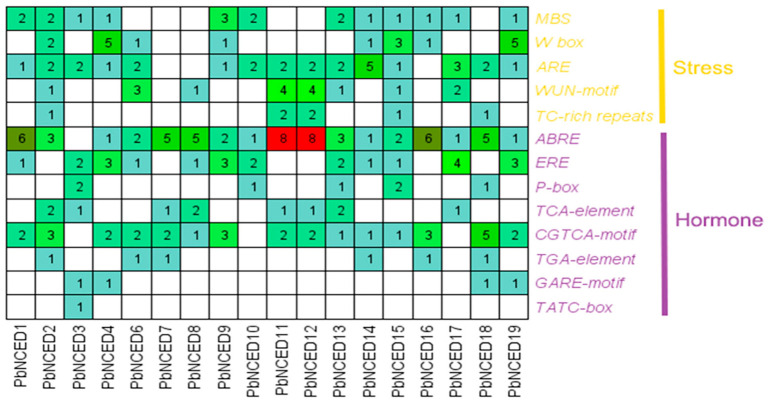
Distribution of stress- and hormone-related *cis*-elements in the promoter regions of *PbNCED* genes. The number represents the number of *cis*-elements.

To investigate the transcriptional regulation and potential functions of *PbNCEDs* in stress responses, the *cis*-elements in the promoter regions (the 2000 bp sequences upstream from the translation start sites) of the *PbNCED* genes were submitted and analyzed in the PlantCARE database (Figure 6). Six hormone-related response elements were displayed in Figure 6, namely, the ABA responsive element (ABRE), ethylene-responsive element (ERE), auxin-responsive elements TGA-element, the gibberellin responsive elements P-box, GARE-motif, and TATC-box, salicylic acid responsive TCA-element, and methyl jasmonate responsive element (CGTCA-motif). The number of ABRE elements was the highest among the hormone-related *cis*-elements. The stress-related response elements included MBS, W-box, ARE, WUN-motif, and TC-rich repeats. The ARE element was found in most of *PbNCED* genes. The *cis*-element analysis illustrated that *PbNCED* genes could respond to various stresses.

### 2.6. Tissue-Specific Expression of PbNCED Genes

**Figure 7:**
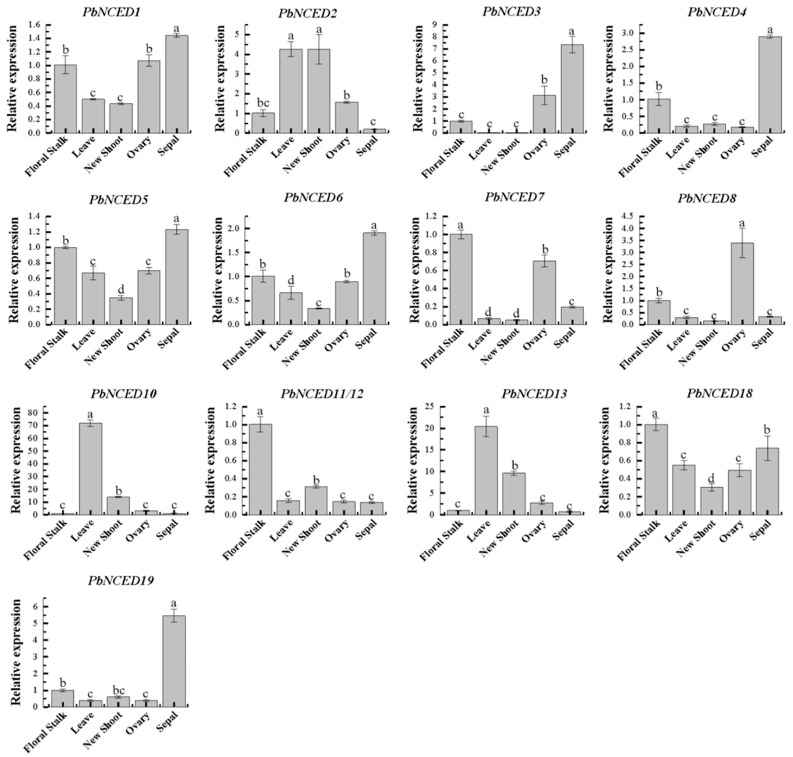
Relative expression level of *PbNCED* genes in different tissues. Different letters indicated significant differences between groups (*p* < 0.05).

To further explain the functions of *PbNCED* genes in the development of ‘Kuerle Xiangli’ (Figure 7), the expression levels of thirteen selected *PbNCED* genes in different tissues (floral stalk, leave, new shoot, ovary, and sepal) were analyzed by qRT-PCR. *PbNCED7*, *PbNCED11*, *PbNCED12*, and *PbNCED18* displayed relatively high expression levels in floral stalk. *PbNCED2* and *PbNCED10* showed high expression levels in leaves and new shoots, respectively. Moreover, *PbNCED1*, *PbNCED3*, *PbNCED4*, *PbNCED5*, *PbNCED6*, and *PbNCED19* were specifically expressed in the sepals. *PbNCED8* was highly expressed in ovaries, compared with other genes. These genes played diverse roles in ‘Kuerle Xiangli’ development.

### 2.7. Effect of Exogenous GA_3_ and PP_333_ on Content of GA_3_, IAA and ABA in ‘Kuerle Xiangli’

In order to better evaluate the effect of exogenous GA_3_ and PP_333_ treatment, the GA_3_, IAA, and ABA content was assessed in the development stages of ovaries and sepals (Table S4). The results showed that the content of GA_3_ was significantly higher in GA_3_ and PP_333_-treated sepals compared with those in the control at 5 days. GA_3_ and ABA content in GA_3_ and PP_333_-treated ovaries decreased after rising, but IAA showed an obvious upward trend, and a significant difference was seen at 9 days compared to control. The content of IAA and ABA was significantly lower than those after PP_333_ treatment, but the GA_3_ content was higher than those after PP_333_ treatment in sepals at 5 days. In addition, the content of IAA and GA_3_ was significantly higher than those after PP_333_ treatment in sepals at 9 days.

The ratio of GA_3_/ABA and IAA/ABA was significantly higher in the GA_3_-treated sepals and ovaries at 5 days and 9 days than in the control. The GA_3_/ABA and IAA/ABA ratio was significantly lower in the PP_333_-treated sepals and ovaries compared to the control at 5 days and 9 days. Interestingly, the ratio of IAA/ABA had the highest value after GA_3_ treatment in sepals on day 5, but the ratio of IAA/ABA was the highest in ovaries on day 9 (Table S5). This result indicated that exogenous GA_3_ and PP_333_ affect the development of ‘Kuerle Xiangli’ flowers by changing the level of endogenous hormones, especially the content of abscisic acid.

### 2.8. Expression Analysis of PbNCED Genes under Exogenous GA_3_ and PP_333_

**Figure 8:**
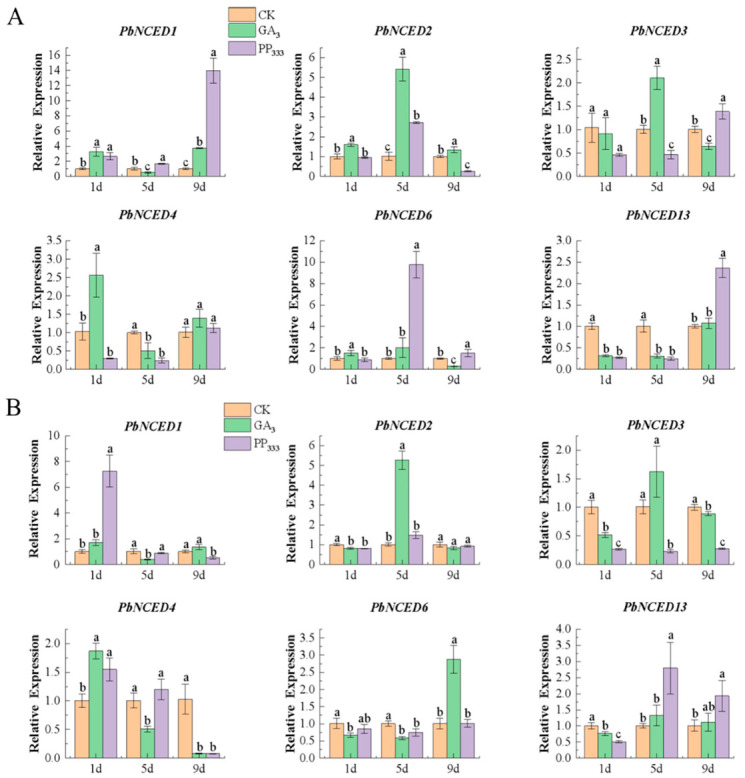
The relative expression levels of the *PbNCEDs* in sepal (**A**) and ovary (**B**). Different lowercase letters indicate significant differences at the level of *p* < 0.05 between treatments.

We randomly selected six *PbNCEDs* to study their expression levels under the effect of exogenous GA_3_ and PP_333_ treatment by using qRT-PCR (Figure 8). The qRT-PCR analysis revealed that the expression levels of *PbNCED2* and *PbNCED3* decreased after rising, while the expression of *PbNCED1* rose after decreasing the GA_3_ treatment. Additionally, the *PbNCED6* had the highest expression level after GA_3_ treatment as compared with the control and PP_333_ treatment in ovaries at 9 days. The expression level of *PbNCED2* decreased after increasing the PP_333_ treatment in ovaries and sepals, and three genes (*PbNCED1*, *PbNCED3*, and *PbNCED13*) exhibited a higher expression level than the control and GA_3_ treatment in sepals at 9 days. The result showed that *PbNCED* genes could respond to hormones.

### 2.9. Correlation Analysis for PbNCED Gene Expresssion Level, Content of GA_3,_ IAA, and ABA in ‘Kuerle Xiangli’ during Sepal and Ovary Development

**Figure 9:**
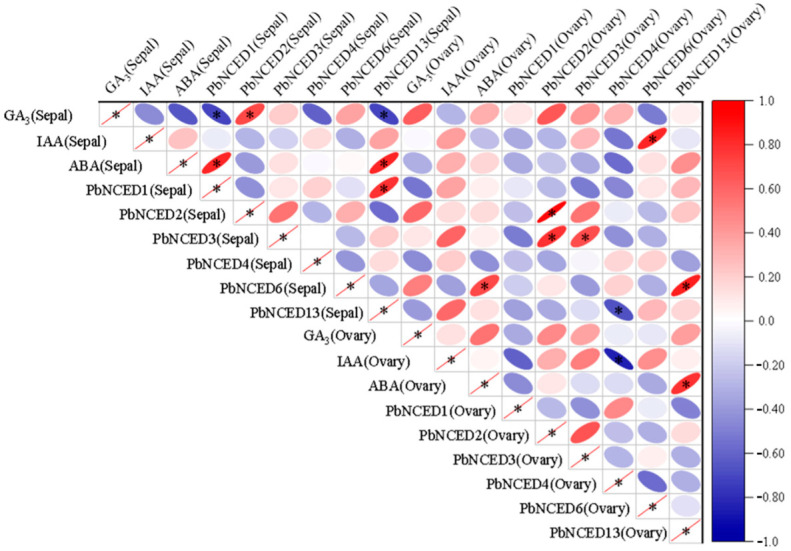
Correlation analysis for biosynthesis gene, content of GA3, IAA, and ABA in ‘Kuerle Xiangli’ during sepal and ovary development. Different asterisk indicated significant differences between groups (* *p* < 0.05).

Correlation analysis between the content of GA_3_, IAA, and ABA in *PbNCED* genes was carried out to explore the correlation between phytohormones and *PbNCED* genes (Figure 9). For this analysis, among the *PbNCED* genes, the expression patterns of *PbNCED1* and *PbNCED13* had a positive correlation with content of ABA, but they had a negative correlation with content of GA_3_ in the sepals. Additionally, the expression pattern of *PbNCED4* had a negative correlation with content of IAA in the ovaries. Taken together, these results indicate that the *PbNCED* gene could affect synthesis of GA_3_, IAA, and ABA in ‘Kuerle Xiangli’ during sepal and ovary development.

## 3. Discussion

In the current study, a total of 19 *PbNCED* genes were identified in pear and those genes were classified into five subfamilies, namely, Group Ⅰ (six), Group Ⅱ (three), Group Ⅲ (five), Group Ⅳ (two), and Group Ⅴ (three). Consistent with a previous study [29], most of the *PbNCED* proteins were localized in the chloroplasts. Motifs of *PbNCED* members’ arrangements were similarities in the same subgroup but obvious differences occur between different subgroups. The result implied that *PbNCED* genes might exhibit similar functions in the same subgroups. Moreover, multiple sequence alignments revealed that the histidine (H) of *Pb*NCED was highly conserved in *PbNCED* sequence, which is similar to previous reports [18]. The *Ka/Ks* ratio analysis indicated that the duplicated *PbNCED* gene pairs evolved under negative selection, consistent with an earlier study [30].

Some valuable clues about the functional role of *PbNCED* genes involved in specific ‘Kuerle Xiangli’ physiological processes were obtained. For example, ABA synthesis played a role in the promotion of leaf senescence through *BnNCED3* as a positive regulator [31]. In Citrus reshni, *CrNCED1* had the highest expression in leaves and the lowest level was detected in the root [32]. The *AhNCED1* transcript and endogenous ABA both accumulate predominantly in peanut leaves in response to dehydration [33]. In our study, *PbNCED10* was specially expressed in leaves, with extremely low expression levels in other tissues. In addition, *PbNCED1*, *PbNCED3*, *PbNCED4*, *PbNCED5*, *PbNCED6*, and *PbNCED19* were specifically expressed in the sepals. In the present study, expression analysis by RT-PCR of *PbNCED* genes implied that those genes had tissue-specific expression.

*Cis*-element played an important role in regulating gene expression such as in response to drought stress and the signal transduction of hormone [34]. In a previous study, *PpNCED1* and *PpNCED5* gene promoted ABA biosynthesis and accelerated cell senescence by activating ROS signals in peach [35]. Overexpression of *VaNCED1* results in increased endogenous ABA accumulation and induced the gene response of JA in grape [36]. Signals of ABA and IAA cross-talk each other [37,38]. ABA negatively controls hypocotyl elongation acting on GA metabolic genes, affecting GA signaling and repressing auxin biosynthetic genes [39]. The ABRE element was the most abundant among the *PbNCED1*, *PbNCED11*, *PbNCED12*, and *PbNCED13*. These genes might have functions similar to those described above. In this study, many hormone-related response elements and stress-related response elements were found, indicating that *PbNCED* plays an important role and has a complex function in the hormone regulation of plant biological processes and the stress response in ‘Kuerle Xiangli’.

In this study, the ratio of GA_3_/ABA and IAA/ABA was GA_3_ treatment > CK > PP_333_ treatment, and they had significant differences to one another. The result showed that there was antagonism between GA_3_ or IAA and ABA. Some valuable clues about the role of IAA (or GA_3_) and ABA in different plants are uncovered. For example, in the development of strawberry fruits, there was synergism between IAA and GA, but IAA (or GA) and ABA had antagonism [40]. The rates of falling flowers and fruit of glycyrrhiza uralensis are negatively correlated with the IAA level and positively correlated with the ABA level [41]. IAA played a major role, while ABA had the opposite effect on stigma exertion in tomato [42]. In maize, the contents of IAA and GA were positively correlated with the maximum seed filling rate, seed weight, and mean filling rate, while ABA was negatively correlated [43]. The phytohormone content assays showed an increase in the content of abscisic acid in young wheat ears, but a decrease in the content of auxin and gibberellins [44]. Additionally, the qRT-PCR method was used to detect the relative expression of *PbNCED* genes in ovaries and sepals after they were sprayed with distilled water, PP_333_, and GA_3_ at full bloom stage. The results showed that the expression of *PbNCED1* and *PbNCED13* gradually increased with the time delay, which was consistent with the ABA content change after GA_3_ and PP_333_ treatment in sepals. We speculated that *PbNCED1* and *PbNCED13* positively regulated the synthesis of ABA in sepals. In addition, the results of correlation analysis further proved the reliability of this speculation. The expression of *PbNCED2* and *PbNCED13* decreased after rising after GA_3_ and PP_333_ treatment in ovaries, consistent with the ABA content change. Further, the expression levels of *PbNCED2* after GA_3_ treatment were significantly higher than those after PP_333_ treatment, but the expression levels of *PbNCED13* were significantly lower than those after PP_333_ treatment in ovaries on 5 d. We speculated that *PbNCED2* positively regulated the synthesis of ABA after GA_3_ treatment in ovaries, while *PbNCED13* positively regulated the synthesis of ABA after PP_333_ treatment in ovaries.

## 4. Materials and Methods

### 4.1. Plant Materials

The plant materials used in this study were selected in spring 2021 at the Shayidong Horticulture Field, Korla, Xinjiang Province. Nine uniform twenty-year-old ‘Kuerle Xiangli’ trees were selected and three trees in each treatment were sprayed with 50 mg/L GA3, 500 mg/L PP333, and water at the bloom stage, respectively. Flowers, mature leaves, and secondary shoots were collected from each tree on the first, fifth, and ninth days of the bloom stage, respectively. After collection, petals, stamens, pistils, and floral shoots, were manually removed, and ovaries and sepals were collected, respectively. All tissues were immediately frozen in liquid N and stored at −80 until use.

### 4.2. Genome-Wide Identification and Protein Properties of NCED Family in Pear

The whole genome of the Pyrus bretschneideri (taxid:225117) was downloaded from NCBI (https://www.ncbi.nlm.nih.gov/, accessed on 20 April 2021). The protein sequences of NCED in *Arabidopsis* were downloaded from TAIR (https://www.arabidopsis.org/index.jsp). These sequences were used to search pear protein sequence data with the BLASTP program (*p* < 1 × 10^−6^). Additionally, to further identify PbNCED candidates, the Hidden Markov Model (HMM) analysis was used for the search. We downloaded HMM profile of RPE65 (PF03055) from the Pfam protein family database (http://pfam.xfam.org/), which was download and used as the query file (*p* < 1 × 10^−6^) to search the pear protein sequence data. After removing the redundant hits, the presence of RPE65 domains was verified by searching the Conserved Domains Database (CDD: https://www.ncbi.nlm.nih.gov/Structure/cdd/cdd.shtml), with ambiguous sequences being manually confirmed using the SMART (http://smart.embl-Heidelberg.de/smart/set_mode.cgi?NORMAL=1).

### 4.3. Sequence Analyses and Phylogenetic Tree Construction

The biochemical features, including molecular weight (MW) and isoelectric point (pI) of PbNCED proteins, were determined by the ExPASy protparam tool (https://web.expasy.org/protparam/). The protein secondary structure of PbNCED proteins was predicted with SOPMA (https://npsa-prabi.ibcp.fr/cgi-bin/npsa_automat.pl?page=npsa_sopma.html). The subcellular localizations of the PbNCED proteins were predicted with WOLF PSORT (http://www.genscript.com/wolfpsort.html). The MEME tool (http://meme-suite.org/tools/meme) was used to predict the motifs of PbNCED proteins with an optimal width of 6-200 residues and the maximum number of motifs set to 15. The domain sequences of the PbNCED proteins aligned using Clustal Omega (https://www.ebi.ac.uk/Tools/msa/clustalo/), and the result was displayed by Jalview software 2.11.2.6 [45]. The exon–intron structures of the PbNCED genes were identified by using the TBtools software [46]. The phylogenetic trees were constructed using the neighbor-joining (NJ) method of MEGA-X with parameters of 1000 bootstrap replicates and pairwise deletion [47]. One additional tree was constructed using the same methodology. Sequences of NCED proteins from rice and grape were downloaded from the Ensemble (http://plants.ensemble.org/species.html).

### 4.4. Gene Duplication and Synteny Analysis

To exhibit the synteny relationship of the orthologous NCED genes from *Arabidopsis*, pear, and apple, the syntenic analysis were examined using TBtools software with default parameters. Analysis of gene duplication events were examined by TBtools software. Non-synonymous (ka) and synonymous (ks) substitution of each duplicated PbNCED genes were calculated using KaKs_Calculator 2.0. The genome sequences of apple were downloaded from the GDR database (https://www.rosaceae.org/).

### 4.5. Interaction Network and Cis-Regulatory Element Analyses

The blastp program was used between the pear NCED proteins and the *Arabidopsis* NCED proteins; each pear NCED protein matched a homologous *Arabidopsis* NCED protein with the highest score. Six *Arabidopsis* NCED proteins, which represent the pear NCED proteins, were uploaded to the String website (https://string-db.org) to predict protein interactions. For promoter analysis, the upstream 2000 bp region of the transcription start site (ATG) for each PbNCED gene was defined from the pear genome and the stress-related and hormone-related *cis*-elements were analyzed by the PlantCARE tool (http://bioinformatics.psb.ugent.be/webtools/plantcare/html/).

### 4.6. Determination of Phytohormones

For GA3, IAA, and ABA extraction, approximately 1 g of ovaries and sepals was weighed and extracted with methanol at 4 °C for 12 h. Each mix was centrifuged for 30 min at 6000 rpm, and the supernatants were then stored at 4 °C. Each precipitate was also extracted with methanol twice. Petroleum ether was added to adsorb phenolics and pigments from the resulting supernatants. Final clean-up of the extract was conducted by extracting with ethyl acetate at pH 3.0, adjusted with formic acid. Finally, the extracts were centrifuged and evaporated, and then HPLC analysis was conducted on the dried samples. Separation of phytohormones (GA3, IAA and ABA) was performed using a ZORBAX Eclipse Plus C18 column (250 mm × 4.6 mm, 5 μm). The mobile phase of GA3 was (A) acetomitrile; and (B) water (with 0.1% (*v*/*v*) formic acid), at a flow rate of 1 mL min^−1^. The mobile phase of IAA was (A) methanol; and (B) water (with 0.1% (*v*/*v*) formic acid), at a flow rate of 0.8 mL min^−1^. The mobile phase of ABA was (A) acetomitrile; and (B) water (with 1.8% (*v*/*v*) acetic acid), at a flow rate of 1 mL min^−1^. 

### 4.7. RNA Extraction and Gene Expression Analysis

The total RNA from pear tissues was extracted using the Plant RNA Extraction Mini Kit (NovaBio, Shanghai, China), according to the manufacturer’s instructions. All RNA were analyzed by agrose gel electrophoresis and then quantified with a Thermo Nano Drop 2000 spectrophotometer. Then, these RNAs were reverse transcribed into first-strand cDNA, following the manufacturer’s procedure for the HyperScriptTM III RT SuperMix for the qPCR with the gDNA Remover Kit (TNovaBio, Shanghai, China). The qRT-PCR primers were designed by Primer3Plus website (http://www.primer3plus.com/cgi-bin/dev/primer3plus.cgi) and are shown in Supplementary Table S6. The quantitative RT-PCR was carried out with the Bio-Rad CFX96 Real Time PCR System. The reaction program was set as follows: 95 °C for 30 s, followed by 42 cycles of 95 °C for 10 s and 60 °C for 30 s. The actin gene was used as the internal reference gene, and each sample was repeated 3 times. The data from real-time PCR amplification was analyzed using the 2^−△△Ct^ method [48]. 

### 4.8. Statistical Analysis 

Figures were drawn using ORIGIN 2020 (Microcal Software, Inc., Northampton, MA, USA). Significant difference analyses (*p* < 0.05) were carried out using one-way ANOVA with Duncan’s test (SPSS 19.0, SPSS Inc., Chicago, IL, USA).

## 5. Conclusions

**Figure 10:**
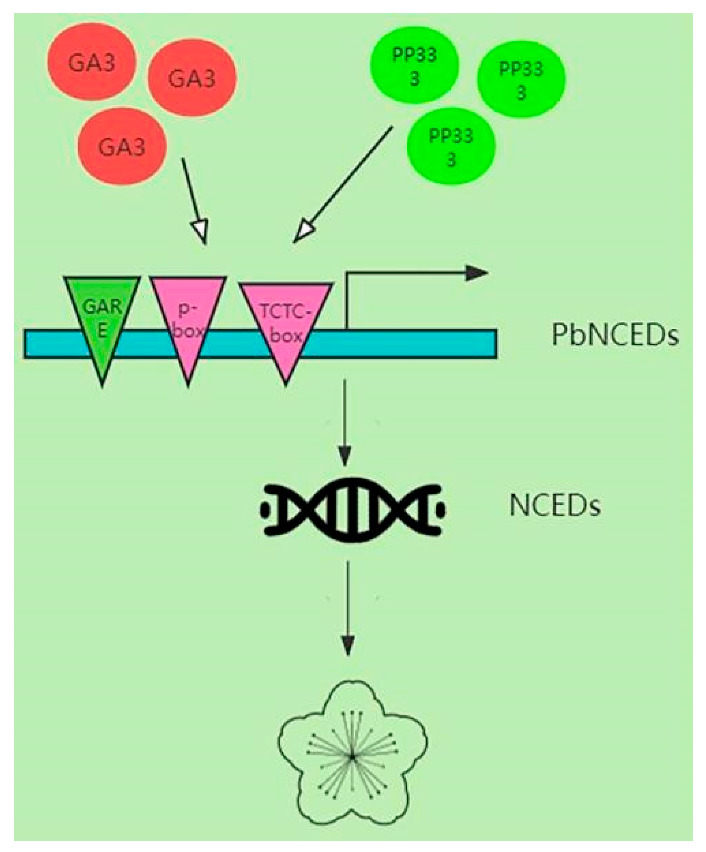
Patterns of calyx abscission and persistent of ‘Kuerle Xiangli’ regulated by exogenous GA_3_ and PP_333_.

In our study, we identified a total of 19 *NCED* genes in the pear genome and analyzed their gene structure, structural domains, and conserved motifs. The phylogenetic tree showed that pear *NCED* genes could be divided into five subgroups. Chromosomal localization and covariance analysis showed that the 19 NCEDs were randomly distributed on 14 scaffolds and had undergone strong purifying selection during evolution. The *PbNCED* genes may have been shown to participate in the regulation of abscission and the persistent calyx after spraying PP_333_ or GA_3_, thus increasing ABA synthesis and carotenoid accumulation (Figure 10). Our findings supply a better understanding of the important genes and regulation pathways related to calyx abscission in ‘Kuerle Xiangli’.

## Data Availability

All data in this study can be found in public databases and supplements, as described in the Section 4 Materials and Methods.

## Supplementary Materials

The supporting information can be downloaded at: https://www.mdpi.com/article/10.3390/ijms24087566/s1.

## References

[1] Raghavendra A.S., Gonugunta V.K., Christmann A., Grill E. (2010). ABA perception and signalling. Trends Plant Sci..

[2] Nonogaki M., Sall K., Nambara E., Nonogaki H. (2014). Amplification of ABA biosynthesis and signaling through a positive feedback mechanism in seeds. Plant J..

[3] Manzi M., Lado J., Rodrigo M.J., Arbona V., Gómez-Cadenas A. (2016). ABA accumulation in water-stressed Citrus roots does not rely on carotenoid content in this organ. Plant Sci..

[4] McAdam S.A.M., Brodribb T.J., Banks J.A., Hedrich R., Atallah N.M., Cai C., Geringer M.A., Lind C., Nichols D.S., Stachowski K. (2016). Abscisic acid controlled sex before transpiration in vascular plants. Proc. Natl. Acad. Sci. USA.

[5] Santosh Kumar V.V., Yadav S.K., Verma R.K., Shrivastava S., Ghimire O., Pushkar S., Rao M.V., Senthil Kumar T., Chinnusamy V. (2021). The abscisic acid receptor OsPYL6 confers drought tolerance to *indica* rice through dehydration avoidance and tolerance mechanisms. J. Exp. Bot..

[6] Iuchi S., Kobayashi M., Taji T., Naramoto M., Seki M., Kato T., Tabata S., Kakubari Y., Yamaguchi-Shinozaki K., Shinozaki K. (2001). Regulation of drought tolerance by gene manipulation of 9-cis-epoxycarotenoid dioxygenase, a key enzyme in abscisic acid biosynthesis in *Arabidopsis*. Plant J..

[7] Seiler C., Harshavardhan V.T., Rajesh K., Reddy P.S., Strickert M., Rolletschek H., Scholz U., Wobus U., Sreenivasulu N. (2011). ABA biosynthesis and degradation contributing to ABA homeostasis during barley seed development under control and terminal drought-stress conditions. J. Exp. Bot..

[8] Schwartz S.H., Tan B.C., Gage D.A., Zeevaart J.A.D., McCarty D.R. (1997). Specific oxidative cleavage of carotenoids by VP14 of maize. Science.

[9] Zhang L., Ma G., Kato M., Yamawaki K., Takagi T., Kiriiwa Y., Ikoma Y., Matsumoto H., Yoshioka T., Nesumi H. (2012). Regulation of carotenoid accumulation and the expression of carotenoid metabolic genes in citrus juice sacs in vitro. J. Exp. Bot..

[10] Kato M. (2012). Mechanism of carotenoid accumulation in citrus fruit. J. Jpn. Soc. Hortic. Sci..

[11] Jia H.-F., Chai Y.-M., Li C.-L., Lu D., Luo J.-J., Qin L., Shen Y.-Y. (2011). Abscisic acid plays an important role in the regulation of strawberry fruit ripening. Plant Physiol..

[12] Jia H.-F., Lu D., Sun J.-H., Li C.-L., Xing Y., Qin L., Shen Y.-Y. (2013). Type 2C protein phosphatase ABI1 is a negative regulator of strawberry fruit ripening. J. Exp. Bot..

[13] Zhang M., Leng P., Zhang G., Li X. (2009). Cloning and functional analysis of 9-*cis*-epoxycarotenoid dioxygenase (NCED) genes encoding a key enzyme during abscisic acid biosynthesis from peach and grape fruits. J. Plant Physiol..

[14] Huang Y., Jiao Y., Xie N., Guo Y., Zhang F., Xiang Z., Wang R., Wang F., Gao Q., Tian L. (2019). *OsNCED5*, a 9-*cis*-epoxycarotenoid dioxygenase gene, regulates salt and water stress tolerance and leaf senescence in rice. Plant Sci..

[15] Bao G., Zhuo C., Qian C., Xiao T., Guo Z., Lu S. (2016). Co-expression of *NCED* and *ALO* improves vitamin C level and tolerance to drought and chilling in transgenic tobacco and stylo plants. Plant Biotechnol. J..

[16] Xia H., Wu S., Ma F. (2014). Cloning and expression of two 9-*cis*-epoxycarotenoid dioxygenase genes during fruit development and under stress conditions from *Malus*. Mol. Biol. Rep..

[17] Zhang T., Gao Y., Han M., Yang L. (2021). Changes in the physiological characteristics of *Panax ginseng* embryogenic calli and molecular mechanism of ginsenoside biosynthesis under cold stress. Planta.

[18] Tan B.-C., Joseph L.M., Deng W.-T., Liu L., Li Q.-B., Cline K., McCarty D.R. (2003). Molecular characterization of the *Arabidopsis* 9-*cis* epoxycarotenoid dioxygenase gene family. Plant J..

[19] Li Q., Yu X., Chen L., Zhao G., Li S., Zhou H., Dai Y., Sun N., Xie Y., Gao J. (2021). Genome-wide identification and expression analysis of the NCED family in cotton (*Gossypium hirsutum* L.). PLoS ONE.

[20] Chernys J.T., Zeevaart J.A. (2000). Characterization of the 9-*cis*-epoxycarotenoid dioxygenase gene family and the regulation of abscisic acid biosynthesis in avocado. Plant Physiol..

[21] Iuchi S., Kobayashi M., Yamaguchi-Shinozaki K., Shinozaki K. (2000). A stress-inducible gene for 9-*cis*-epoxycarotenoid dioxygenase involved in abscisic acid biosynthesis under water stress in drought-tolerant cowpea. Plant Physiol..

[22] Gan Z., Shan N., Fei L., Wan C., Chen J. (2019). Isolation of the 9-*cis*-epoxycarotenoid dioxygenase (*NCED*) gene from kiwifruit and its effects on postharvest softening and ripening. Sci. Hortic..

[23] Wang X., Liu F., Shi X., Wang X., Ji X., Wang Z. (2019). Evolution and expression of *NCED* family genes in *Vitis vinifera*. Chin. Bull. Bot..

[24] Chevreau E., Skirvin R.M., Abu-Qaoud H.A., Korban S.S., Sullivan J.G. (1989). Adventitious shoot regeneration from leaf tissue of three pear (*Pyrus* sp.) cultivars in vitro. Plant Cell Rep..

[25] Ma L., Zhou L., Quan S., Xu H., Yang J., Niu J. (2019). Integrated analysis of mRNA-seq and miRNA-seq in calyx abscission zone of Korla fragrant pear involved in calyx persistence. BMC Plant Biol..

[26] Wu J., Wang Z., Shi Z., Zhang S., Ming R., Zhu S., Khan M.A., Tao S., Korban S.S., Wang H. (2013). The genome of the pear (*Pyrus bretschneideri* Rehd.). Genome Res..

[27] Klepikova A.V., Kasianov A.S., Gerasimov E.S., Logacheva M.D., Penin A.A. (2016). A high resolution map of the *Arabidopsis thaliana* developmental transcriptome based on RNA-seq profiling. Plant J..

[28] Auldridge M.E., Block A., Vogel J.T., Dabney-Smith C., Mila I., Bouzayen M., Magallanes-Lundback M., DellaPenna D., McCarty D.R., Klee H.J. (2006). Characterization of three members of the *Arabidopsis* carotenoid cleavage dioxygenase family demonstrates the divergent roles of this multifunctional enzyme family. Plant J..

[29] Pei X., Wang X., Fu G., Chen B., Nazir M.F., Pan Z., He S., Du X. (2021). Identification and functional analysis of 9-*cis*-epoxy carotenoid dioxygenase (NCED) homologs in *G. hirsutum*. Int. J. Biol. Macromol..

[30] Priya R., Siva R. (2015). Analysis of phylogenetic and functional diverge in plant nine-*cis* epoxycarotenoid dioxygenase gene family. J. Plant Res..

[31] Xu P., Cai W. (2017). Functional characterization of the *BnNCED3* gene in *Brassica napus*. Plant Sci..

[32] Xian L., Sun P., Hu S., Wu J., Liu J.-H. (2014). Molecular cloning and characterization of *CrNCED1*, a gene encoding 9-*cis*-epoxycarotenoid dioxygenase in *Citrus reshni*, with functions in tolerance to multiple abiotic stresses. Planta.

[33] Wan X.-R., Li L. (2006). Regulation of ABA level and water-stress tolerance of *Arabidopsis* by ectopic expression of a peanut 9-*cis*-epoxycarotenoid dioxygenase gene. Biochem. Biophys. Res. Commun..

[34] Wittkopp P.J., Kalay G. (2011). Cis-regulatory elements: Molecular mechanisms and evolutionary processes underlying divergence. Nat. Rev. Genet..

[35] Wang P., Lu S., Zhang X., Hyden B., Qin L., Liu L., Bai Y., Han Y., Wen Z., Xu J. (2021). Double NCED isozymes control ABA biosynthesis for ripening and senescent regulation in peach fruits. Plant Sci..

[36] Ju Y.-L., Min Z., Yue X.-F., Zhang Y.-L., Zhang J.-X., Zhang Z.-Q., Fang Y.-L. (2014). Overexpression of grapevine *VvNAC08* enhances drought tolerance in transgenic *Arabidopsis*. Plant Physiol. Biochem..

[37] Wang L., Hua D., He J., Duan Y., Chen Z., Hong X., Gong Z. (2011). *Auxin Response Factor2* (*ARF2*) and its regulated homeodomain gene *HB33* mediate abscisic acid response in *Arabidopsis*. PLoS Genet..

[38] Zhao Y., Xing L., Wang X., Hou Y.-J., Gao J., Wang P., Duan C.-G., Zhu X., Zhu J.-K. (2014). The ABA receptor PYL8 promotes lateral root growth by enhancing MYB77-dependent transcription of auxin-responsive genes. Sci. Signal..

[39] Lorrai R., Boccaccini A., Ruta V., Possenti M., Costantino P., Vittorioso P. (2018). Abscisic acid inhibits hypocotyl elongation acting on gibberellins, DELLA proteins and auxin. AoB Plants.

[40] Kim J., Lee J.G., Hong Y., Lee E.J. (2019). Analysis of eight phytohormone concentrations, expression levels of ABA biosynthesis genes, and ripening-related transcription factors during fruit development in strawberry. J. Plant Physiol..

[41] Yan B., Hou J., Cui J., He C., Li W., Chen X., Li M., Wang W. (2019). The Effects of Endogenous Hormones on the Flowering and Fruiting of *Glycyrrhiza uralensis*. Plants.

[42] Cheng M.-Z., Gong C., Zhang B., Qu W., Qi H.-N., Chen X.-L., Wang X.-Y., Zhang Y., Liu J.-Y., Ding X.-D. (2021). Morphological and anatomical characteristics of exserted stigma sterility and the location and function of *SlLst* (*Solanum lycopersicum* Long styles) gene in tomato. Theor. Appl. Genet..

[43] Ahmad S., Kamran M., Zhou X., Ahmad I., Meng X., Javed T., Iqbal A., Wang G., Su W., Wu X. (2021). Melatonin improves the seed filling rate and endogenous hormonal mechanism in grains of summer maize. Physiol. Plant..

[44] Zhang W., Wang J., Huang Z., Mi L., Xu K., Wu J., Fan Y., Ma S., Jiang D. (2019). Effects of Low Temperature at Booting Stage on Sucrose Metabolism and Endogenous Hormone Contents in Winter Wheat Spikelet. Front. Plant Sci..

[45] Procter J.B., Carstairs G.M., Soares B., Mourão K., Ofoegbu T.C., Barton D., Lui L., Menard A., Sherstnev N., Roldan-Martinez D. (2021). Alignment of Biological Sequences with Jalview. Mult. Seq. Alignment.

[46] Chen C., Chen H., Zhang Y., Thomas H.R., Frank M.H., He Y., Xia R. (2020). TBtools: An Integrative Toolkit Developed for In-teractive Analyses of Big Biological Data. Mol. Plant.

[47] Kumar S., Stecher G., Li M., Knyaz C., Tamura K. (2018). MEGA X: Molecular Evolutionary Genetics Analysis across Computing Platforms. Mol. Biol. Evol..

[48] Livak K.J., Schmittgen T.D. (2001). Analysis of relative gene expression data using real-time quantitative PCR and the 2^−ΔΔCT^ method. Methods.

