# Supplementary material for: Genome-Wide Identification and Expression Analysis of NCED Gene Family in Pear and Its Response to Exogenous Gibberellin and Paclobutrazol
Source: Int J Mol Sci. 2023 Apr 20;24(8):7566. doi: 10.3390/ijms24087566 (PMC10144387; doi:10.3390/ijms24087566)
Supplement: Supplementary file 1 [file ijms-24-07566-s001.zip › Supplementary table.pdf]

Table S1 Information of Physicochemical properties of NCED gene family members

Gene name	Gene ID	Scaff	Scaff rename	Deduced protein		
				Size of aa	MW(kD)	pI
PbNCED1	XM_009356579.2	NW_008988076.1	Scaff2	613	68.37	6.63
PbNCED2	XM_009362625.2	NW_008988119.1	Scaff3	603	66.64	6.45
PbNCED4	XM_009366657.2	NW_008988157.1	Scaff5	583	65.03	5.72
PbNCED5	XM_009366793.1	NW_008988158.1	Scaff6	474	53.92	5.88
PbNCED6	XM_009366794.2	NW_008988158.1	Scaff6	508	57.54	6.40
PbNCED7	XM_009367200.2	NW_008988163.1	Scaff7	601	66.04	7.31
PbNCED8	XM_009367201.2	NW_008988163.1	Scaff7	601	66.12	7.64
PbNCED9	XM_009369180.2	NW_008988186.1	Scaff8	563	62.21	6.68
PbNCED10	XM_009369225.2	NW_008988186.1	Scaff8	615	69.13	5.61
PbNCED11	XM_009369487.2	NW_008988189.1	Scaff9	617	68.67	6.65
PbNCED12	XM_009369488.2	NW_008988189.1	Scaff9	617	68.67	6.65
PbNCED13	XM_009369742.2	NW_008988191.1	Scaff10	615	69.13	5.61
PbNCED14	XM_009374927.1	NW_008988263.1	Scaff11	616	68.38	5.99
PbNCED15	XM_009378252.2	NW_008988325.1	Scaff12	584	65.28	6.10
PbNCED16	XM_018645641.1	NW_008989098.1	Scaff13	517	58.04	5.63
PbNCED17	XM_018646301.1	NW_008989585.1	Scaff14	569	64.34	5.63
PbNCED18	XM_018647118.1	NW_008988075.1	Scaff1	555	62.63	6.16
PbNCED19	XM_018649309.1	NW_008988157.1	Scaff5	583	65.06	5.81

Table S2 Secondary structure of NCED family proteins

Gene name	Alpha helix/%	Beta turn/%	Extended strand/%	Random coil/%	Subcellular localization
PbNCED1	17.13	6.36	21.53	54.98	Chloroplast
PbNCED2	15.26	5.80	24.05	54.89	Chloroplast
PbNCED3	17.14	6.49	54.24	22.13	Chloroplast
PbNCED4	14.92	6.35	56.60	22.13	Chloroplast
PbNCED5	13.71	6.54	55.49	24.26	Peroxisome
PbNCED6	12.20	5.31	59.65	22.83	Cytoplasmic
PbNCED7	15.31	5.82	57.07	21.80	Chloroplast
PbNCED8	14.64	5.99	57.24	22.13	Chloroplast
PbNCED9	17.94	6.75	52.22	23.09	Cytoplasmic
PbNCED10	20.00	4.72	54.96	20.33	Chloroplast；Cytoplasmic
PbNCED11	14.91	5.02	58.67	21.39	Chloroplast
PbNCED12	14.91	5.02	58.67	21.39	Chloroplast
PbNCED13	20.00	4.72	54.96	20.33	Chloroplast；Cytoplasmic
PbNCED14	17.69	6.01	54.71	21.59	Chloroplast
PbNCED15	17.64	6.16	54.11	22.09	Chloroplast
PbNCED16	13.54	6.00	56.87	23.60	Mitochondria
PbNCED17	11.25	5.98	60.81	21.97	Cytoplasmic
PbNCED18	20.90	6.31	48.47	24.32	Peroxisome；Cytoplasmic
PbNCED19	15.27	5.66	57.29	21.78	Chloroplast

Table S3 Analysis of duplication time of Ka/Ks values response of the homologous gene pairs for NCED gene family in pear

Homologous gene pair	Ka	Ks	Ka/Ks	Duplication time (millions years ago)
PbNCED1 & PbNCED11	0.0394	0.2550	0.1547	84.984
PbNCED1 & PbNCED12	0.0394	0.2550	0.1547	84.984
PbNCED5 & PbNCED18	0.0337	0.1313	0.2566	43.763
PbNCED6 & PbNCED18	0.0349	0.0942	0.3707	31.402
PbNCED4 & PbNCED19	——	——	——	——

Table S4 Effects of different treatments on concentrations of hormones in sepal and ovary of '*Pyrus sinkiangensis* Yu'.

Time (d)	Treatment	GA_3 ($ng \cdot g^{-1}$)		IAA ($ng \cdot g^{-1}$)		ABA ($ng \cdot g^{-1}$)	
		Sepal	Ovary	Sepal	Ovary	Sepal	Ovary
1	CK	535.98±9.94a	313.25±10.61a	762.70±9.28a	242.13±12.29a	116.68±3.70a	539.29±9.24a
	GA_3	364.83±12.18b	284.44±7.78b	565.73±9.99b	226.67±10.64a	107.86±7.88a	193.07±6.48b
	PP_{333}	532.35±11.36a	298.37±10.00ab	534.09±10.32c	196.57±6.08b	115.70±4.21a	175.40±7.07b
5	CK	505.15±11.36c	639.66±11.38a	764.90±12.36a	260.70±11.45a	189.14±5.89b	616.29±7.89b
	GA_3	698.20±11.67a	574.27±11.47b	591.54±13.60c	274.40±12.68a	128.98±4.89c	415.75±8.22c
	PP_{333}	592.34±6.91b	619.39±12.02a	634.52±12.63b	210.25±7.55b	311.99±4.17a	845.74±7.44a
9	CK	404.82±12.77a	360.43±10.02b	977.15±18.79b	223.88±7.47b	509.78±7.98b	230.99±4.98b
	GA_3	302.04±11.84b	407.45±6.99a	1127.92±17.94a	280.81±11.48a	312.08±7.47c	174.47±6.24c

PP_{333}	231.56±11.62c	239.39±10.64c	680.53±9.99c	277.03±12.29a	927.53±9.38a	552.69±6.29a

Data are mean ± standard error. Significant differences at P < 0.05 are denoted with different letters.

Table S5 Effects of different treatments on the balance between hormones in sepal and ovary of '*Pyrus sinkiangensis* Yu'.

Time(d)	Treatment	GA3/ABA		IAA/ABA	
		Sepal	Ovary	Sepal	Ovary
1	CK	6.546±0.287a	0.449±0.015c	4.601±0.231a	0.581±0.01c
	GA3	5.280±0.479b	1.174±0.016b	3.393±0.139b	1.474±0.009a
	PP333	4.626±0.257a	1.121±0.011a	4.604±0.069b	1.706±0.126b
5	CK	4.046±0.062b	0.423±0.024b	2.672±0.032b	1.038±0.005b
	GA3	4.589±0.069a	0.660±0.017a	5.418±0.115a	1.381±0.002a
	PP333	2.035±0.068c	0.249±0.007c	1.899±0.048c	0.732±0.011c
9	CK	1.917±0.007b	0.969±0.011b	0.794±0.013b	1.560±0.01b
	GA3	3.615±0.029a	1.609±0.009a	0.967±0.015a	2.337±0.044a
	PP333	0.734±0.003c	0.501±0.017c	0.250±0.01c	0.433±0.016c

Data are mean ± standard error. Significant differences at P < 0.05 are denoted with different letters.

Table S6 Additional file 1 Primer sequences for quantitative real time PCR

Gene	Forward primer (5'-3')	Reverse primer (5'-3')
PbNCED1	CTACAAGACACCGCCACCTT	CAAGTTCCATTGTTGCGGCA
PbNCED2	CGGAGAGATGATCACGGGTG	TTTCGGACAGCACGCTTTTG
PbNCED3	CCTCGACGGAGCCTACATTC	ATTGGCCTGTAAAGACCCGG
PbNCED4	CTACAGCCCCGTGTTTCCAT	GTCAACTGCGTACCTGGGAA
PbNCED5	TGGGCAATGGGTCTGTCAAA	GGCCTTGGACATTTCCTCCA
PbNCED6	CATGCATGACCCTGTACCGA	AAACAACTTCGTCCCCCTCC
PbNCED7	GCTTGGTAGACAGCTCACGT	GTACTTGAGGTGCGGCTTCT
PbNCED8	CATGGACCCACCGGATTCAA	AGCAATACCCGAACACCTGG
PbNCED10	GGTGTCTGGGGAAGTTGAGG	TCCTGATCCCCAGATGGTGT
PbNCED11	TCAGCCAAGAACGTGACCTC	GGTCCCCTGTTTCGGTGATT
PbNCED12	TCAGCCAAGAACGTGACCTC	GGTCCCCTGTTTCGGTGATT
PbNCED13	GGTGTCTGGGGAAGTTGAGG	TCCTGATCCCCAGATGGTGT
PbNCED18	AGCAATGGTGGATGTCGAGG	AAGCACTCAGGAAGATGGCC
PbNCED19	GCGAGCTGATACATGGCTCT	GAGAGGATATCCGGCTTCGC
Actin	CCATCCAGGCTGTTCTCTC	GCAAGGTCCAGACGAAGG
